# Predictive Factors of Positive Circumferential and Longitudinal Margins in Early T3 Colorectal Cancer Resection

**DOI:** 10.1155/2020/6789709

**Published:** 2020-06-27

**Authors:** M. Ashraf Balbaa, Noha Elkady, Emad M. Abdelrahman

**Affiliations:** ^1^General Surgery Department, Faculty of Medicine, Menoufia University, Menoufia 32511, Egypt; ^2^Pathology Department, Faculty of Medicine, Menoufia University, Menoufia 32511, Egypt; ^3^General Surgery Department, Faculty of Medicine, Benha University, Benha 13511, Egypt

## Abstract

**Background:**

Malignant involvement of circumferential resection margin (CRM) and longitudinal resection margin (LRM) after surgical resection of colorectal cancer (CRC) are associated with higher rates of recurrence and development of distant metastasis. This can influence the overall patient's prognosis. The aim of the current study was to identify pathological factors as predictors for the involvement of resection margins in early T3 CRC. *Patients and Methods.* Fifty patients radiologically diagnosed to have cT3a/b (CRC) were included in the study. After resection, the pathological examination was performed to identify patients with positive CRM and/or LRM. Relations between the different pathological parameters and the CMR and LRM involvements were assessed.

**Results:**

Positive CRM was present in 17 cases (34%), while positive LRM was found in 6 cases (12%). The involvement of both margins was significantly associated with rectal tumors and tumors with infiltrative gross appearance, grade III, deeper invasion, and positive lymph node metastases. Also, there was a significant association between both margins' positivity and other pathological parameters as signet ring carcinoma, tumor budding, perineural and vascular invasion, high microvessel density (MVD), and sinusoidal vascular pattern, while the presence of necrosis and infiltrative advancing tumor front was significantly associated with CRM involvement only. The depth of tumor invasion and signet ring carcinoma were identified as independent predictor factors for positive CRM and LRM, respectively.

**Conclusion:**

Preoperative identification of these pathological parameters can be a guide to tailor the management plan accordingly.

## 1. Introduction

Globally, Colorectal Cancer (CRC) is the third most commonly diagnosed cancer and the second cancer-related leading cause of death [1]. In Egypt, it occupies the 7^th^ among all cancers, where it represents 3.47% and 3% of cancers in males and females, respectively [2]. Currently, the treatment strategy for CRC patients involves a multimodal approach based on tumor-related characteristics and patient-related factors [3]. However, surgery remains the mainstay curative treatment for patients with nonmetastatic CRC and the quality of surgical procedure can significantly influence both short- and long-term disease outcomes [4]. One of the crucial pillars of surgical quality is achieving negative resection margins. Negative circumferential resection margin (CRM) as well as longitudinal resection margin (LRM) can be considered the hallmark of a successful oncologic resection [5]. Many studies have demonstrated that CRM involvement is able to predict local recurrence and poor prognosis among patients with rectal as well as colon cancer [5–7]. On the other hand, the LRM positivity has been shown to be a predictor for local recurrence, development of distant metastasis, and decreased disease-free survival [8–10]. In spite of confining to standard surgical rules to achieve grossly negative resection margin (R0), still, positive resection margins are detected microscopically on postoperative histopathological examination [5, 11, 12]. This can be clearly demonstrated in early T3 tumors where surgery is the main line of therapy. For colon cancer, guidelines recommend neoadjuvant therapy to be used only in selected cases of T4 and not for T3 tumors [13, 14]. For rectal cancer, although NCCN guidelines [15] recommend neoadjuvant therapy for T3 tumors, still ESMO guidelines [16] recommend neoadjuvant therapy for tumors > cT3b as a routine therapy and for cT3a/b tumors in conditioned indications [17]. We assume that pathological features indicating rapidly dividing, infiltrative, and aggressive tumors have an association with positive margins. The aim of this study was to explore the pathological factors as predictors for the involvement of resection margins of early T3 CRC. If these factors can be recognized preoperatively, intraoperative modulation of surgical techniques and/or the addition of other therapeutic modalities can be applied.

## 2. Patients and Methods

This study included 50 radiologically selected patients to be cT3a/b tumors out of 196 cases of operable CRC that were not candidates for neoadjuvant therapy. The patients have been operated upon at Surgery Departments of the Main Hospitals of Menoufia and Benha Universities, during the period from January 2016 to May 2019. An approval to conduct the research was obtained from both institutes' ethical and research committees (No# 12/2015 SURG 7 and 0134-12/15). A written informed consent was obtained from all included patients. Exclusion criteria included patients with locally advanced tumors with evidence of local infiltration to other organs or surrounding tissue cT3 c/d or T4 or those who have been operated upon in emergency situations, as perforated or obstructed cases. Metastatic cases and operable ones after receiving neoadjuvant therapy were excluded as well. Detailed history has been obtained from all patients. Systematic physical examination was performed followed by full preoperative investigations, including colonoscopy and biopsy as well as complete metastatic workup. All biopsies were histologically confirmed to be CRC. MRI for rectal cancer was performed to select patients with cT3a/b. Spiral CT for colon cancer was performed to select T3 tumors that extend to the pericolic tissue but not to adjacent organs. It was demonstrated as thickening and infiltration of pericolic fat. Surgical resection was performed to all cases after thorough intraoperative assessment of the nonmetastatic stage of the tumors. Surgical resection included right hemicolectomy, left hemicolectomy, sigmoidectomy, anterior resection, and abdominoperineal resection. Colonic resection was performed with at least 5 cm longitudinal resection margins with excision of the adjoining mesentery that harbors all the tumor-draining lymph nodes. Proximal ligation of the arterial supply of the resected portion was performed to ensure harvesting all the draining lymph nodes with subsequent removal of adjoining devascularized bowel by this ligation. Circumferential resection included resection of the retroperitoneal adventitial tissue of the cecum, ascending, or descending colon. For the rectal resection, at least 2 cm of grossly free distal margin was obtained. Due to the proximal high ligation of the inferior mesenteric artery, the proximal longitudinal margin was very abundant. The principles of total mesorectal excision were followed to ensure grossly free CRM of the rectum by sharp and precise dissection at the anatomical fascial planes. Great care has been taken to avoid injury of the hypogastric nerve.

Labeling and orientation of the specimens were performed before sending to the Pathology Department at the Faculty of Medicine, Menoufia University. Surgical specimens were grossly examined to assess tumor site, size, and gross appearance. Hematoxylin and Eosin (H&E) stained slides were histologically examined using light microscopy to confirm the diagnosis. Identification of different tumor pathological findings was performed, including histopathological type, grade, depth of invasion, and lymph node involvement. Special tumor characters had been evaluated as the presence of tumor-associated inflammation, desmoplasia, budding, necrosis, mitotic and apoptotic indices, perineural and vascular invasion, microvessel density (MVD), vascular pattern, and the pattern of advancing tumor front. Special attention was paid to determine CRM and LRM involvement. The circumferential margin was defined as the shortest distance measured from the microscopically deepest area of tumor infiltration to the stained CRM. Positive CRM involvement was defined as tumor presence in a distance ≤1 mm from the nonperitonealized surface of resection or by serosal penetration of the peritonealized portions of the colon [18]. The LRM was defined as the distance from the tumor edge to the closest resection margin(s). Resection margin of 2 cm was considered adequate [18].

According to the involvement CRM, patients were divided into two groups (CRM-positive and CRM-negative groups). The same was performed according to LRM involvement (LRM-positive and LRM-negative groups). Relations between the different pathological findings and the CMR and LRM involvements were assessed.

Statistical analysis was performed using SPSS-20 (Statistical Package for Social Sciences version 20). Univariate analysis was performed to identify significant predictors of a positive CRM and positive LRM. Qualitative parameters were expressed as the frequency with percentage rates and the Chi-square test was used to assess the statistically significant association. On the other hand, quantitative parameters were expressed as a range (minimum and maximum), mean, and standard deviation where Student's *t*-test and Mann–Whitney *U* test were used to assess the statistical significance. The crude odds ratios (OR) and their 95 percent confidence intervals (95% CI) were calculated for each variable. Pathological parameters associated with positive CRM and those with positive LRM with a *P* value <0.05 were included in a multivariate logistic regression to identify those variables that are independently associated with either positive CRM or positive LRM, respectively.

## 3. Results

The mean age of the included patients was 63.8 ± 4.1 years with more incidence in males (31 cases; 62%) than females (19 cases; 38%). Forty-one (82%) cases were diagnosed as colon cancer while 9 (18%) cases were rectal cancer.

Twenty-three cases were grossly fungating type (46%). Adenocarcinoma represented almost half of the cases (26 cases; 52%) and the others were either mucinous (15 cases; 30%) or signet ring carcinoma (9 cases; 18%). Positive CRM was present in 17 cases (34%), while positive LRM was found in 6 cases (12%) (Table 1).

**Figure 1:**
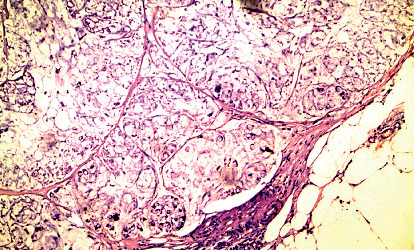
Positive circumferential margin in the case of mucoid carcinoma with signet ring differentiation (H&E 200).

**Figure 2:**
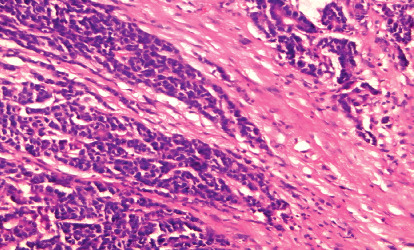
Advancing tumor front (invasive type) in the case of high-grade adenocarcinoma (H&E 200).

The study showed that positive CRM was significantly associated with rectal location (*P*=0.004), infiltrative gross pattern (*P*=0.005), signet ring carcinoma (*P*=0.002), deeper tumor invasion (*P* < 0.001) (Figure 1), grade III tumors (*P*=0.034), invasive pattern of advancing tumor front (*P*=0.002) (Figure 2), positive lymph node metastasis (*P*=0.001), tumor budding (*P*=0.016), presence of necrosis (*P*=0.029), perineural and vascular invasion (*P*=0.04 and 0.021), high MVD (*P* < 0.001), and presence of sinusoidal vascular pattern (*P*=0.001) (Table 2).

**Figure 3:**
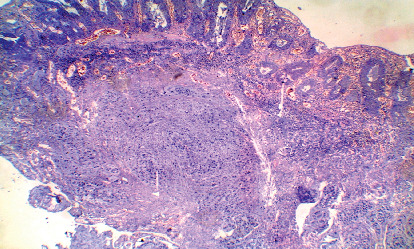
Signet ring carcinoma associated with positive longitudinal margin and creeping malignant cells beneath the intestinal glands (H&E 200).

**Figure 4:**
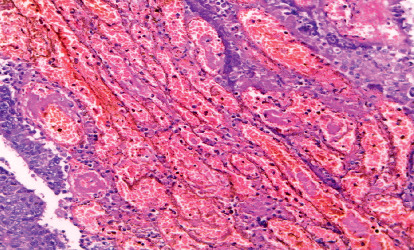
High-grade invasive adenocarcinoma associated with sinusoidal vascular pattern (H&E 200).

On the other hand, the study showed a significant association between positive LRM and rectal location (*P*=0.007), infiltrative gross pattern (*P*=0.049), signet ring carcinoma (*P* < 0.001) (Figure 3), deeper tumor invasion (*P*=0.021), grade III tumors (*P*=0.042), positive lymph nodes involvement (*P*=0.01), tumor budding (*P*=0.018), perineural and vascular invasion (*P*=0.009 and 0.011), high MVD (0.004), and sinusoidal vascular pattern (*P*=0.001) (Figure 4) (Table 2).

Table 3 shows the univariate analysis of the different pathological parameters and their relations with both positive CRM and LRM. Multivariate logistic regression revealed that invasion of pericolorectal tissue/serosa was the independent predictor factor for positive CMR (*P* < 0.001), with the marginal significance of infiltrative gross pattern (*P*=0.055), while signet ring type was the independent predictor factor for positive LRM (*P*=0.035) with the marginal significance of high MVD (*P*=0.53) and sinusoidal vascular pattern (*P*=0.54) (Table 4).

## 4. Discussion

Presence of gross or microscopic evidence of malignant tumor at the resection margins of CRC specimen is a universally poor prognostic factor [19]. Previous studies have concentrated on the CRM of the rectum as a very strong predictor of tumor recurrence. In a meta-analysis that included over 17,000 patients, Nagtegaal and Quirke [20] were able to demonstrate that involvement of CRM was a strong predictor of local recurrence (HR 2.7, 95% CI 1.7–4.3), distant metastases (HR 2.8, 95% CI 1.9–4.3), and survival as well (HR 1.7, 95% CI 1.3–2.3). On the other hand, for the colon, LRM had great attention in research neglecting the significance of its radial margin. As demonstrated by Amri et al. [5], a cohort of nearly 1000 patients was essential to have enough statistical power to show the consequences of positive CRM of colon cancer. Believing in the significance of both margins, CRM and LRM as predictors of the patient's outcome, the current study has explored different pathological factors that influence the positivity of both resection margins in both colon and rectum.

The key for the optimal CRM is the respect to the embryonic fascia by total mesorectal excision for rectal cancer [21] and resection of the retroperitoneal adventitial soft tissue of the partially peritonealized colon [5]. Bujko et al. [22] demonstrated in their review that subclinical distal bowel intramural spread is present within 1 cm distally from visible tumor edge in a considerable proportion of patients. Consequently, for patients who are undergoing anterior resection for low-lying cancer, a distal bowel clear margin of at least >1 cm is minimally acceptable. On the other hand, Hohenberger et al. [23] concluded in their study that the standard LRM in colon cancer should be at least 5 cm on both sides of the tumor. Confining to these standards was performed during surgical resection in the current study.

Aggressive tumors are associated with uncontrolled cell proliferation and extensive invasion and metastasis. Uncontrolled proliferation is due to the activation of cell cycle genes and the loss of apoptosis-inducing ones and is reflected histologically as high mitotic and low apoptotic indices. While the ability for invasion is due to oncological metaplasticity and epithelial-mesenchymal transition where the cells lose adhesion and acquire cytoskeleton reorganization, contractility, and invadopodia and then become capable of stromal invasion. The advancing tumor front is one of the determinants of tumor invasion and in aggressive tumors; it is usually an invasive pattern [24].

Positivity of the resection margins has been shown to be influenced by a lot of factors as tumor location, stage, grade, lymph node metastases, positive vascular and perineural invasion, and pattern of advancing tumor front [5, 11, 12, 25]. The previously mentioned factors have been demonstrated in the current study, in addition to other pathological parameters that were significantly associated with positive margins, such as signet ring carcinoma, tumor budding, necrosis, high MVD, and ectatic vascular pattern. The selection of higher “T” of the included cases within the present study may explain the encountered slightly higher rates of positive CRM (34%) and LRM (12%) when compared with the previously reported prevalence in similar studies. Positive rates of CRM were reported to be 5.3% by Armi et al. [5], 17.6% by Kang et al. [26], 22% by Eriksen et al., and 28% by Birbeck et al. [27], while rates of positive LRM were reported to be 1.5% by Zeng et al. [10], 6.83% by Orosco et al. [28], and 7.9% by Kanters et al. [25].

In the current study, multivariate analysis has demonstrated that deeper tumor invasion up to the pericolorectal tissue/serosa was an independent predictor of positive CRM. This observation is matching with Rickles et al. [11] and Warrier et al. [12] who demonstrated the significant relation between tumor “T” depth of invasion and positive CRM. The infiltrative gross pattern of tumors has been shown to have marginal significance as an independent predictor as well. Although the results did not reach the statistical threshold of significance, it seems that both parameters are coincident, as infiltrating tumor pattern is directly related to the depth of tumor invasion.

In the previous studies [29, 30], it has been established that mucinous and signet ring types of CRC have a worse prognosis compared to other varieties of CRC. They are characterized by being prevalent in more advanced stages of the disease, with a much higher rate of lymphatic metastasis, serous infiltration, and peritoneal dissemination. In addition, these two types of carcinomas have higher rates of the local extension, which leads to a lesser chance for curative resection and decreases the overall survival rate [31]. Signet ring carcinomas are considered high-grade adenocarcinomas. In these tumors, there is a loss of E-cadherin, cell adherence, tight junctions, and cell-cell interaction with the acquisition of stem cell-like characteristics leading to enhanced tumor growth, invasion, and metastasis [32, 33]. Obviously, the mucin provides pressure on the bowel wall with more tendency for tumor extension. On the other hand, the intracellular mucin display may induce swelling of the tumor cells, due to its ability to imbibe water, and allow them to pass through the bowel layers with further dissemination [34]. This coincides with our observations as it has been shown that signet ring carcinoma was an independent predictor for positive LRM in colorectal cancer. In a study by Rickles et al. [11], they have demonstrated that signet ring cell carcinoma and mucinous adenocarcinoma are independent factors for CRM involvement.

Neovascularization is an important factor in cancer growth and metastasis because it is involved in the transport of various nutrients to the tumor cells [35]. Vascular changes in tumor areas are due to mediators secreted by tumor cells or the surrounding microenvironment. The vascular patterns are either capillary-like or sinusoid-like vessels which form a cobweb-like network and facilitate tumor invasion and metastasis [36]. Microvessel density (MVD) has been documented to have a prognostic value in colon cancer [37]. In the current study, MVD has been shown to have a marginal significance to be an independent predictor for positivity of the of LRM. The limited number of included cases could be a factor that influenced the results and the statistical power threshold of significance could have been reached if the number of patients was quite larger. In the literature, we could not trace a similar model correlating the neovascularization with positive involvement of CRC resection margins. However, in a study by Tarta et al. [38], they documented a significant association between tumor high microvessel count and its depth of invasion. In another study by Mohamed et al. [39], they documented a significant correlation between MVD and pathological stage of the tumor and with vascular invasion which has an influence on tumor depth of invasion. Consequently, the depth of invasion has its impact on CRM as discussed earlier.

Although the determined predictor factors cannot be avoided or modified, they have the advantage of being identifiable preoperatively before the layout of the treatment plan. Due to enhancing diagnostic accuracy, especially in pelvic MRI [40], preoperative threatened CRM was regarded as an essential indication for neoadjuvant chemoradiation for rectal cancer to reduce CRM-positive rates [5, 26]. Preoperative anticipation of positive resection margins dictates the necessity for wider resection with the possibility of the use of intraoperative radiation therapy as well [28]. Although neoadjuvant chemotherapy has been demonstrated as a treatment strategy in locally advanced rectal cancer, it was not established to be a treatment option in operable locally advanced colon cancer. In 2012, the FOxTROT trial [41] was the first randomized study in assessing preoperative chemotherapy in locally advanced operable colon cancer that came up with the feasibility of the regimen with acceptable toxicity and perioperative morbidity. The same concept has been explored by other authors and concluded that this regimen can now be considered as a treatment option in locally advanced colon cancer that can induce marked histological downstaging and a halving of the rate of incomplete resections with improving surgical outcomes [42–44]. These evidences may offer an additional option for treatment in colon cancers at risk as well.

## 5. Conclusions

The depth of the tumor and signet ring type are independent predictor factors for positive CRM and LRM, respectively, in early T3 CRC. Preoperative identification of these parameters can help in the modulation of the treatment plan. Inclusion of neoadjuvant therapy and performing a wider margin of resection during surgery should be considered in cases of positive independent predictors. Further study has to be performed with a larger number of included patients to determine the actual role of the marginal significant independent predictors.

## Figures and Tables

**Table 1:** Distribution of the studied cases according to different clinicopathological parameters (*n* = 50).

	No. (%)
Sex	
Male	31 (62%)
Female	19 (38%)
Age (years)	
Median (min.–max.)	63 (49–72)
Mean ± SD	63.8 ± 4.1
Site	
Colon	41 (82%)
Rectum	9 (18%)
Size	
Median (min.–max.)	4 (3–6)
Mean ± SD	4.3 ± 1
Gross appearance	
Ulcer	15 (30%)
Infiltrating	12 (24%)
Fungating	23 (46%)
Histopathological type	
Adenocarcinoma	26 (52%)
Mucinous	15 (30%)
Signet ring	9 (18%)
Depth	
Pericolorectal tissue/serosa	20 (40%)
Muscularis propria	30 (60%)
Advancing tumor front	
Invasive pattern	37 (74%)
Broad pushing margin (cohesive pattern)	13 (26%)
Desmoplasia	20 (40%)
Tumor budding	18 (36%)
Lymph nodes involvement	16 (32%)
Inflammation	27 (54%)
Necrosis	19 (38%)
Perineural invasion	5 (10%)
Vascular invasion	10 (20%)
Tumor grade	
G1	15 (30%)
GII	17 (34%)
GIII	18 (36%)
Mitotic index	
Median (min.–max.)	8 (1–11)
Mean ± SD	6.4 ± 3.1
Apoptotic index	
Median (min.–max.)	9 (2–15)
Mean ± SD	7.7 ± 3.7
MVD	
Median (min.–max.)	8 (2–18)
Mean ± SD	7.7 ± 3.4
Sinusoidal vascular pattern	
Negative	39 (78%)
Positive	11 (22%)
Positive longitudinal resection margin	6 (12%)
Positive circumferential resection margin	17 (34%)

**Table 2:** Relation between circumferential and longitudinal resection margins and different clinicopathological parameters (*n* = 50).

	Circumferential resection margin		Test of sig.	*P*	Longitudinal resection margin		Test of sig.	*P*
	Negative (*n* = 33)	Positive (*n* = 17)			Negative (*n* = 44)	Positive (*n* = 6)		
Sex								
Male	20 (60.6%)	11 (64.7%)	*χ* ^2^ = 0.080	0.777	27 (61.4%)	4 (66.7%)	*χ* ^2^ = 0.063	1.000
Female	13 (39.4%)	6 (35.3%)			17 (38.6%)	2 (33.3%)		
Age (years)								
Median (Min.–max.)	63 (49–72)	66 (55–70)	*t* = 0.507	0.615	63 (49–72)	63.5 (55–67)	*t* = 0.792	0.432
Mean ± SD.	63.5 ± 4.2	64.2 ± 4			63.9 ± 4.1	62.5 ± 4.5		
Tumor site								
Colon	31 (93.9%)	10 (58.8%)	*χ* ^2^ = 9.374^*∗*^	0.004^*∗*^	39 (88.6%)	2 (33.3%)	*χ* ^2^ = 10.941^*∗*^	0.007^*∗*^
Rectum	2 (6.1%)	7 (41.2%)			5 (11.4%)	4 (66.7%)		
Tumor size								
Median (min.–max.)	4 (3–6)	4 (3–6)	*t* = 0.376	0.709	4 (3–6)	4.5 (3–6)	*t* = 0.886	0.380
Mean ± SD.	4.3 ± 1	4.4 ± 0.9			4.3 ± 0.9	4.7 ± 1.2		
Gross appearance								
Ulcer	9 (27.3%)	6 (35.3%)	*χ* ^2^ = 10.448^*∗*^	0.005^*∗*^	14 (31.8%)	1 (16.7%)	*χ* ^2^ = 5.470^*∗*^	0.049^*∗*^
Infiltrating	4 (12.1%)	8 (47.1%)			8 (18.2%)	4 (66.7%)		
Fun gating	20 (60.6%)	3 (17.6%)			22 (50%)	1 (16.7%)		
Histopathological type								
Adenocarcinoma	23 (69.7%)	3 (17.6%)	*χ* ^2^ = 12.624^*∗*^	0.002^*∗*^	25 (56.8%)	1 (16.7%)	*χ* ^2^ = 13.543^*∗*^	<0.001^*∗*^
Mucinous	7 (21.2%)	8 (47.1%)			15 (34.1%)	0 (0%)		
Signet ring	3 (9.1%)	6 (35.3%)			4 (9.1%)	5 (83.3%)		
Depth								
Pericolorectal tissue/serosa	3 (9.1%)	17 (100%)	FE	<0.001^*∗*^	15 (34%)	5 (83.3%)	*χ* ^2^ = 5.35^*∗*^	0.021^*∗*^
Muscularis propria	30 (90.9%)	0 (0%)			29 (66%)	1 (16.7%)		
Tumor grade								
I	13 (39.4%)	2 (11.8%)	*χ* ^2^ = 6.742^*∗*^	0.034^*∗*^	15 (34.1%)	0 (0%)	*χ* ^2^ = 5.685^*∗*^	0.042^*∗*^
II	12 (36.4%)	5 (29.4%)			16 (36.4%)	1 (16.7%)		
III	8 (24.2%)	10 (58.8%)			13 (29.5%)	5 (83.3%)		
Advancing tumor front								
Invasive pattern	20 (60.6%)	17 (100%)	*χ* ^2^ = 9.050^*∗*^	0.002^*∗*^	31 (70.5%)	6 (100%)	*χ* ^2^ = 2.396	0.122
Broad pushing margin (cohesive pattern)	13 (39.4%)	0 (0%)			13 (29.5%)	0 (0%)		
Lymph nodes involvement	5 (15.2%)	11 (64.7%)	*χ* ^2^ = 12.662^*∗*^	0.001^*∗*^	11 (25%)	5 (83.3%)	*χ* ^2^ = 8.257^*∗*^	0.010^*∗*^
Inflammation	15 (45.5%)	12 (70.6%)	*χ* ^2^ = 2.853	0.091	23 (52.3%)	4 (66.7%)	*χ* ^2^ = 0.440	0.674
Desmoplasia	11 (33.3%)	9 (52.9%)	*χ* ^2^ = 1.797	0.180	16 (36.4%)	4 (66.7%)	*χ* ^2^ = 2.020	0.202
Tumor budding	8 (24.2%)	10 (58.8%)	*χ* ^2^ = 5.824	0.016^*∗*^	13 (29.5%)	5 (83.3%)	*χ* ^2^ = 6.630^*∗*^	0.018^*∗*^
Necrosis	9 (27.3%)	10 (58.8%)	*χ* ^2^ = 4.741^*∗*^	0.029^*∗*^	15 (34.1%)	4 (66.7%)	*χ* ^2^ = 2.378	0.184
Perineural invasion	1 (3%)	4 (23.5%)	*χ* ^2^ = 5.239^*∗*^	0.040^*∗*^	2 (4.5%)	3 (50%)	*χ* ^2^ = 12.121^*∗*^	0.009^*∗*^
Vascular invasion	3 (9.1%)	7 (41.2%)	*χ* ^2^ = 7.219^*∗*^	0.021^*∗*^	6 (13.6%)	4 (66.7%)	*χ* ^2^ = 9.280^*∗*^	0.011^*∗*^
Mitotic index								
Median (min.–max.)	7 (1–11)	8 (2–10)	*U* = 265.50	0.620	7 (1–11)	7.5 (4–10)	*U* = 97.50	0.311
Mean ± SD	6 ± 3.2	6.4 ± 2.9			5.9 ± 3.1	7.5 ± 2.3		
Apoptotic index								
Median (min.–max.)	7 (2–13)	10 (2–15)	*U* = 207.50	0.132	9 (2–15)	7.5 (3–11)	*U* = 124.0	39.0
Mean ± SD	7.2 ± 3.5	8.7 ± 4.1			7.8 ± 3.7	7.2 ± 3.9		
MVD								
Median (min.–max.)	7 (2–13)	11 (7–18)	*U* = 88.0^*∗*^	<0.001^*∗*^	7 (2–18)	11 (10–11)	*U* = 39.0^*∗*^	0.004^*∗*^
Mean ± SD	6.3 ± 3	10.2 ± 2.5			7.3 ± 3.4	10.7 ± 0.5		
Sinusoidal vascular pattern								
Negative	29 (87.9%)	10 (58.8%)	*χ* ^2^ = 5.520^*∗*^	0.001^*∗*^	38 (86.4%)	1 (16.7%)	*χ* ^2^ = 14.947^*∗*^	0.001^*∗*^
Positive	4 (12.1%)	7 (41.2%)			6 (13.6%)	5 (83.3%)		

*χ* ^2^: Chi-square test; *t*: Student's *t*-test; *U*: Mann–Whitney test; FE: Fisher's Exact test; *P*: *P* value for association between negative and positive; ^*∗*^: statistically significant at *P* < 0.05.

**Table 3:** Univariate analysis for the clinicopathological parameters affecting longitudinal and circumferential margins (*n* = 50).

	Longitudinal resection margin		Circumferential resection margin	
	*P*	OR (95% CI)	*P*	OR (95% CI)
Sex (male)	0.802	1.259 (0.208–7.638)	0.777	1.192 (0.353–4.018)
Age (years)	0.427	0.924 (0.761–1.123)	0.607	1.039 (0.897–1.204)
Tumor site (rectum)	0.005^*∗*^	15.60^*∗*^ (2.251–108.12)	0.007^*∗*^	10.850^*∗*^ (1.932–60.930)
Tumor size	0.376	1.513 (0.605–3.781)	0.702	1.128 (0.609–2.088)
Gross appearance (infiltrating)	0.021^*∗*^	9.0^*∗*^ (1.398–57.944)	0.010^*∗*^	6.444^*∗*^ (1.567–26.506)
Histopathological type (signet ring)	0.001^*∗*^	50.0^*∗*^ (4.626–540.444)	0.032^*∗*^	5.455^*∗*^ (1.159–25.662)
Tumor grade (Grade III)	0.030^*∗*^	11.923 (1.266–112.287)	0.019^*∗*^	4.464 (1.277–15.608)
Depth (pericolorectal tissue/Serosa)	0.030^*∗*^	11.923^*∗*^ (1.266–112.29)	<0.001^*∗*^	75.0 (11.291–498.196)
Advancing tumor front	0.098	0.152 (0.016–1.411)	0.998	—
Desmoplasia	0.174	3.50 (0.576–21.282)	0.184	2.250 (0.680–7.442)
Tumor budding	0.030^*∗*^	11.923^*∗*^ (1.266–112.29)	0.019^*∗*^	4.464^*∗*^ (1.277–15.608)
Lymph nodes involvement	0.018^*∗*^	15.0^*∗*^ (1.576–142.724)	0.001^*∗*^	10.267^*∗*^ (2.592–40.669)
Inflammation	0.511	1.826 (0.303–11.020)	0.097	2.880 (0.827–10.034)
Necrosis	0.143	3.867 (0.634–23.585)	0.033^*∗*^	3.810^*∗*^ (1.110–13.070)
Vascular invasion (positive)	0.009^*∗*^	12.667^*∗*^ (1.888–84.965)	0.013^*∗*^	7.0^*∗*^ (1.515–32.333)
Mitotic index	0.251	1.220 (0.869–1.713)	0.626	1.050 (0.863–1.277)
Apoptotic index	0.705	0.956 (0.759–1.205)	0.171	1.125 (0.950–1.333)
MVD (high)	0.042^*∗*^	1.401 (1.012–1.940)	0.001^*∗*^	1.731 (1.234–2.429)
Sinusoidal vascular pattern	0.003^*∗*^	31.667^*∗*^ (3.133–320.06)	0.025^*∗*^	5.075^*∗*^ (1.223–21.065)

OR: Odd`s ratio; CI: confidence interval; #: all variables with *P* < 0.05 were included in the multivariate; ^*∗*^: statistically significant at *P* ≤ 0.05.

**Table 4:** Multivariate logistic regression (Forward: Wald) for circumferential and longitudinal resection margins.

		B	SE	Sig.	OR
Circumferential resection margin	Gross appearance (infiltrating)	2.371	1.285	0.055	10.713
	Depth (pericolorectal tissue/serosa)	4.590	1.172	<0.001^*∗*^	98.460

Longitudinal resection margin	MVD (high)	0.679	0.351	0.053	1.972
	Vascular pattern (sinusoidal)	3.780	1.961	0.054	43.820
	Histopathological type (signet ring)	4.684	2.216	0.035^*∗*^	108.229

B: unstandardized coefficients; OR: odds ratio; CI: confidence interval; LL: lower limit.

## Data Availability

Detailed used data during the current study are available from the corresponding author on reasonable request.
